# 4-Phenyl­diazenyl-2-[(*R*)-(1-phenyl­ethyl)imino­meth­yl]phenol

**DOI:** 10.1107/S1600536810007762

**Published:** 2010-03-06

**Authors:** Yoshikazu Aritake, Yoshimasa Watanabe, Takashiro Akitsu

**Affiliations:** a Department of Chemistry, Faculty of Science, Tokyo University of Science, 1-3 Kagurazaka, Shinjuku-ku, Tokyo 162-8601, Japan

## Abstract

The title chiral photochromic Schiff base compound, C_21_H_19_N_3_O, was synthesized from (*R*)-1-phenyl­ethyl­amine and the salicylaldehyde of an azobenzene derivative. The mol­ecule corresponds to the phenol–imine tautomer, the C=N and N—C bond distances being 1.279 (3) and 1.477 (3) Å, respectively. An intra­molecular O—H⋯N hydrogen bond occurs. The diazenyl group adopts a *trans* form with an N=N distance of 1.243 (3) Å.

## Related literature

For applications of Schiff base–metal complexes and azobenzene, see: Akitsu & Einaga (2005*a* ▶,*b* ▶); Akitsu (2007 ▶); Akitsu & Itoh (2010 ▶). For Schiff base ligands, see: Akitsu *et al.* (2004 ▶, 2006 ▶); Miura *et al.* (2009 ▶); Hadjoudis & Mavridis (2004 ▶). For Schiff base compounds with an azobenzene group, see: Aslantaş *et al.* (2007 ▶); Khandar & Rezvani (1999 ▶). 
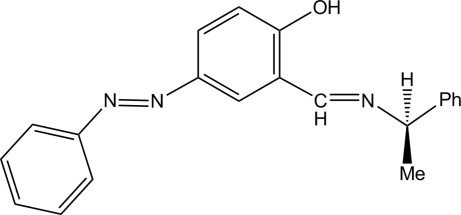


## Experimental

### 

#### Crystal data

C_21_H_19_N_3_O
*M* *_r_* = 329.39
Monoclinic, 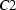

*a* = 22.430 (2) Å
*b* = 5.9566 (6) Å
*c* = 13.4670 (13) Å
β = 106.396 (1)°
*V* = 1726.1 (3) Å^3^
*Z* = 4
Mo *K*α radiation
μ = 0.08 mm^−1^
*T* = 173 K
0.31 × 0.12 × 0.09 mm

#### Data collection

Bruker APEXII CCD diffractometer
Absorption correction: multi-scan (*SADABS*; Sheldrick, 1996 ▶) *T*_min_ = 0.976, *T*_max_ = 0.993
4632 measured reflections
1960 independent reflections
1741 reflections with *I* > 2σ(*I*)
*R*_int_ = 0.029

#### Refinement

*R*[*F* ^2^ > 2σ(*F* ^2^)] = 0.040
*wR*(*F* ^2^) = 0.123
*S* = 0.96
1960 reflections
231 parameters
1 restraint
H atoms treated by a mixture of independent and constrained refinement
Δρ_max_ = 0.26 e Å^−3^
Δρ_min_ = −0.27 e Å^−3^

### 

Data collection: *APEX2* (Bruker, 1998 ▶); cell refinement: *SAINT* (Bruker, 1998 ▶); data reduction: *SAINT*; program(s) used to solve structure: *SHELXS97* (Sheldrick, 2008 ▶); program(s) used to refine structure: *SHELXL97* (Sheldrick, 2008 ▶); molecular graphics: *SHELXTL* (Sheldrick, 2008 ▶); software used to prepare material for publication: *SHELXTL*.

## Supplementary Material

Crystal structure: contains datablocks global, I. DOI: 10.1107/S1600536810007762/zq2030sup1.cif

Structure factors: contains datablocks I. DOI: 10.1107/S1600536810007762/zq2030Isup2.hkl

Additional supplementary materials:  crystallographic information; 3D view; checkCIF report

## Figures and Tables

**Table 1:** Hydrogen-bond geometry (Å, °)

*D*—H⋯*A*	*D*—H	H⋯*A*	*D*⋯*A*	*D*—H⋯*A*
O1—H1⋯N1	1.01 (4)	1.66 (4)	2.557 (3)	145 (3)

**Fig. 1.**
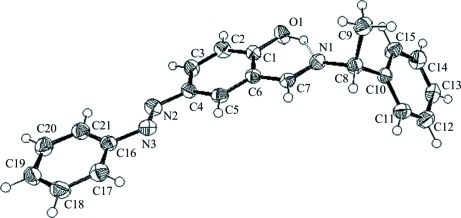
The molecular structure of (I), showing the atom labeling scheme. Displacement ellipsoids are drawn at the 50% probability level.

## supplementary crystallographic information

### Comment

In the recent years, we have developed organic-inorganic hybrid materials of photochromic compounds and chiral Schiff base metal complexes. Due to structural flexibility of such complexes, *cis-trans* photoisomerization of azobenzene could switch conformation of chiral ligands (Akitsu & Einaga, 2005*a,b*). Moreover, multifunctional (photochemical) development of Schiff base Cu^II^, Ni^II^, or Zn^II^ complexes (Akitsu, 2007) and optical anisotropy in polymer films (Akitsu & Itoh, 2010) have been reported so far. On the other hand, free Schiff base ligands may act as photochromic, thermochromic, and fluorescence materials (Hadjoudis *et al.*, 2004). We have developed chiral Schiff base liagnds to include chiral functions (Akitsu *et al.*, 2004, 2006*b*; Miura *et al.*, 2009). Herein we prepared the title chiral photochromic Schiff base compound (I), having two photochromic moieties.

The crystal structure of (I) is similar to that of the analogous Schiff base ligands (Akitsu *et al.*, 2006*b*; Miura *et al., *2009). The molecule of (I) (Fig. 1) adopts an *E* configuration with respect to the imine C=N double bond with C6—C7—N1—C8 torsion angle of 179.21 (16)°. Thus, the π-conjugated system around the imine group is essentially planar. The C1—O1 bond distance of 1.337 (3) Å suggests that it is in the phenol-imine tautomer. The contraction of the C7=N1 bond is also in agreement with the phenol-imine tautomer. As for the azobenzene moiety, the azo N=N double bond adopts an *E* configuration with the N=N distance of 1.243 (3) Å. Beside them, geometric parameters reported agree with the corresponding values for analogous azobenzene derivatives (Aslantaş *et al.*, 2007; Khandar & Rezvani,1999).

The planarity of (I) is stabilized by an intramolecular O—H···N hydrogen bond (Table 1). However, there is no intermolecular hydrogen bonds in the crystal structure.

### Experimental

Treatment of aniline (6.24 g, 67.0 mmol) in 30 ml of 6M HCl and NaNO_2_ (4.69 g, 68.0 mmol) in 30 ml of H_2_O for 30 min at 278 K gave rise to the yellow precursor. Treatment of the precursor and salicylaldehyde (8.18 g, 67.0 mmol) in 30 ml of 10 % NaOH aqueous solution for 1 hour at 278 K, and the resulting brown precipitates were filtrated and washed with water and ethanol, and dried in a desiccator for several days. Treatment of the brown precipitates (0.226 g, 1.00 mmol) and (*R*)-1-phenylethylamine (0.121 g, 1.00 mmol) for 2 hours at 313 K gave rise to redish violet crystals after evaporation (yield 0.21 g, 64 %).

### Refinement

All H atoms were placed at the calculated positions (C-H = 0.95-1.00 Å) and were included in the refinement in the riding mode with *U*_iso_(H) = 1.2-1.5*U*_eq_(C). The OH hydrogen atom was located in a difference Fourier map, and was freely refined. Friedel pairs were merged.

### Crystal data

**Table:**

C_21_H_19_N_3_O	*F*(000) = 696
*M**_r_* = 329.39	*D*_x_ = 1.268 Mg m^−^^3^
Monoclinic, *C*2	Mo *K*α radiation, λ = 0.71073 Å
Hall symbol: C 2y	Cell parameters from 1707 reflections
*a* = 22.430 (2) Å	θ = 2.8–25.6°
*b* = 5.9566 (6) Å	µ = 0.08 mm^−^^1^
*c* = 13.4670 (13) Å	*T* = 173 K
β = 106.396 (1)°	Prism, red violet
*V* = 1726.1 (3) Å^3^	0.31 × 0.12 × 0.09 mm
*Z* = 4	

### Data collection

**Table:**

Bruker APEXII CCD diffractometer	1960 independent reflections
Radiation source: fine-focus sealed tube	1741 reflections with *I* > 2σ(*I*)
graphite	*R*_int_ = 0.029
Detector resolution: 8.333 pixels mm^-1^	θ_max_ = 26.6°, θ_min_ = 1.6°
φ and ω scans	*h* = −16→28
Absorption correction: multi-scan (*SADABS*; Sheldrick, 1996)	*k* = −6→7
*T*_min_ = 0.976, *T*_max_ = 0.993	*l* = −16→16
4632 measured reflections	

### Refinement

**Table:**

Refinement on *F*^2^	Primary atom site location: structure-invariant direct methods
Least-squares matrix: full	Secondary atom site location: difference Fourier map
*R*[*F*^2^ > 2σ(*F*^2^)] = 0.040	Hydrogen site location: inferred from neighbouring sites
*wR*(*F*^2^) = 0.123	H atoms treated by a mixture of independent and constrained refinement
*S* = 0.96	*w* = 1/[σ^2^(*F*_o_^2^) + (0.1*P*)^2^] where *P* = (*F*_o_^2^ + 2*F*_c_^2^)/3
1960 reflections	(Δ/σ)_max_ = 0.001
231 parameters	Δρ_max_ = 0.26 e Å^−^^3^
1 restraint	Δρ_min_ = −0.27 e Å^−^^3^

### Special details

**Table:**

Geometry. All esds (except the esd in the dihedral angle between two l.s. planes) are estimated using the full covariance matrix. The cell esds are taken into account individually in the estimation of esds in distances, angles and torsion angles; correlations between esds in cell parameters are only used when they are defined by crystal symmetry. An approximate (isotropic) treatment of cell esds is used for estimating esds involving l.s. planes.
Refinement. Refinement of *F*^2^ against ALL reflections. The weighted *R*-factor wR and goodness of fit *S* are based on *F*^2^, conventional *R*-factors *R* are based on *F*, with *F* set to zero for negative *F*^2^. The threshold expression of *F*^2^ > σ(*F*^2^) is used only for calculating *R*-factors(gt) etc. and is not relevant to the choice of reflections for refinement. *R*-factors based on *F*^2^ are statistically about twice as large as those based on *F*, and *R*- factors based on ALL data will be even larger.

### Fractional atomic coordinates and isotropic or equivalent isotropic displacement parameters (Å^2^)

**Table:**

	*x*	*y*	*z*	*U*_iso_*/*U*_eq_	
O1	0.99357 (8)	−0.0345 (3)	0.86831 (13)	0.0407 (4)	
C5	1.01961 (10)	0.3842 (4)	0.68547 (16)	0.0311 (5)	
H5	1.0052	0.5273	0.6577	0.037*	
C2	1.06244 (11)	−0.0381 (4)	0.76572 (17)	0.0358 (5)	
H2	1.0775	−0.1809	0.7930	0.043*	
C6	0.99411 (9)	0.2834 (4)	0.75769 (15)	0.0286 (5)	
C4	1.06632 (10)	0.2751 (4)	0.65393 (16)	0.0328 (5)	
C3	1.08666 (11)	0.0649 (4)	0.69370 (18)	0.0364 (5)	
H3	1.1179	−0.0097	0.6710	0.044*	
C17	1.10112 (11)	0.8454 (4)	0.44134 (19)	0.0386 (6)	
H17	1.0665	0.9222	0.4526	0.046*	
C7	0.94575 (9)	0.3980 (4)	0.79195 (15)	0.0286 (5)	
H7	0.9314	0.5415	0.7646	0.034*	
C16	1.11935 (10)	0.6407 (4)	0.48836 (16)	0.0326 (5)	
C1	1.01590 (10)	0.0684 (4)	0.79815 (16)	0.0301 (5)	
C21	1.17050 (11)	0.5300 (4)	0.47323 (18)	0.0382 (6)	
H21	1.1831	0.3894	0.5057	0.046*	
C18	1.13343 (12)	0.9384 (5)	0.37779 (18)	0.0439 (6)	
H18	1.1205	1.0778	0.3444	0.053*	
C13	0.72251 (11)	−0.0416 (5)	0.85593 (19)	0.0414 (6)	
H13	0.6892	−0.1453	0.8464	0.050*	
C10	0.82061 (9)	0.2628 (4)	0.88270 (16)	0.0297 (5)	
C8	0.87446 (9)	0.4249 (4)	0.89349 (16)	0.0311 (5)	
H8	0.8598	0.5583	0.8481	0.037*	
C20	1.20294 (11)	0.6245 (5)	0.41107 (19)	0.0449 (7)	
H20	1.2382	0.5495	0.4010	0.054*	
C19	1.18438 (11)	0.8291 (5)	0.36290 (18)	0.0451 (7)	
H19	1.2068	0.8937	0.3197	0.054*	
C11	0.76386 (10)	0.3026 (5)	0.81026 (17)	0.0371 (5)	
H11	0.7580	0.4359	0.7699	0.045*	
C15	0.82718 (10)	0.0696 (4)	0.94228 (19)	0.0380 (6)	
H15	0.8656	0.0397	0.9923	0.046*	
C14	0.77806 (11)	−0.0812 (5)	0.9297 (2)	0.0441 (6)	
H14	0.7829	−0.2114	0.9720	0.053*	
C12	0.71552 (11)	0.1493 (5)	0.79616 (19)	0.0429 (6)	
H12	0.6773	0.1764	0.7449	0.051*	
C9	0.90390 (11)	0.5033 (5)	1.00386 (17)	0.0393 (6)	
H9A	0.9363	0.6144	1.0046	0.059*	
H9B	0.9224	0.3746	1.0469	0.059*	
H9C	0.8720	0.5714	1.0312	0.059*	
N3	1.08366 (9)	0.5577 (3)	0.55470 (14)	0.0347 (5)	
N1	0.92269 (8)	0.3059 (3)	0.85861 (14)	0.0320 (4)	
N2	1.09774 (9)	0.3622 (3)	0.58460 (15)	0.0375 (5)	
H1	0.9594 (15)	0.061 (7)	0.882 (2)	0.075 (10)*	

### Atomic displacement parameters (Å^2^)

**Table:**

	*U*^11^	*U*^22^	*U*^33^	*U*^12^	*U*^13^	*U*^23^
O1	0.0506 (10)	0.0315 (9)	0.0472 (9)	0.0084 (8)	0.0254 (8)	0.0107 (8)
C5	0.0337 (11)	0.0279 (11)	0.0295 (10)	−0.0018 (10)	0.0053 (8)	0.0015 (10)
C2	0.0434 (12)	0.0261 (12)	0.0387 (11)	0.0061 (10)	0.0127 (10)	0.0024 (10)
C6	0.0303 (10)	0.0268 (11)	0.0271 (9)	−0.0028 (10)	0.0053 (8)	−0.0019 (9)
C4	0.0353 (11)	0.0351 (12)	0.0275 (10)	−0.0048 (11)	0.0082 (8)	0.0000 (10)
C3	0.0405 (12)	0.0336 (13)	0.0382 (12)	0.0049 (11)	0.0163 (10)	−0.0010 (10)
C17	0.0388 (12)	0.0350 (14)	0.0396 (11)	−0.0016 (11)	0.0071 (10)	−0.0017 (10)
C7	0.0272 (10)	0.0272 (11)	0.0296 (10)	0.0010 (9)	0.0052 (8)	0.0010 (9)
C16	0.0344 (11)	0.0363 (13)	0.0247 (10)	−0.0067 (10)	0.0045 (8)	−0.0021 (9)
C1	0.0347 (11)	0.0273 (12)	0.0288 (10)	−0.0020 (10)	0.0096 (9)	−0.0010 (9)
C21	0.0404 (12)	0.0343 (13)	0.0381 (11)	0.0001 (10)	0.0081 (10)	0.0027 (11)
C18	0.0491 (14)	0.0378 (14)	0.0388 (12)	−0.0066 (12)	0.0028 (11)	0.0108 (11)
C13	0.0331 (12)	0.0411 (14)	0.0537 (14)	−0.0038 (11)	0.0183 (10)	−0.0094 (12)
C10	0.0293 (10)	0.0315 (12)	0.0289 (10)	0.0045 (9)	0.0091 (8)	−0.0001 (9)
C8	0.0310 (11)	0.0288 (12)	0.0342 (10)	0.0047 (9)	0.0101 (9)	0.0027 (9)
C20	0.0381 (13)	0.0555 (18)	0.0429 (13)	−0.0007 (13)	0.0141 (11)	−0.0023 (13)
C19	0.0448 (14)	0.0546 (17)	0.0343 (11)	−0.0163 (13)	0.0082 (10)	0.0037 (12)
C11	0.0385 (12)	0.0374 (13)	0.0329 (10)	0.0042 (11)	0.0061 (9)	0.0021 (11)
C15	0.0313 (11)	0.0360 (13)	0.0440 (12)	0.0043 (11)	0.0061 (10)	0.0078 (11)
C14	0.0469 (14)	0.0335 (14)	0.0539 (14)	0.0034 (11)	0.0178 (11)	0.0072 (12)
C12	0.0332 (12)	0.0501 (16)	0.0417 (12)	0.0046 (12)	0.0046 (10)	−0.0059 (12)
C9	0.0361 (12)	0.0402 (14)	0.0402 (12)	0.0025 (11)	0.0083 (10)	−0.0053 (11)
N3	0.0364 (10)	0.0347 (11)	0.0315 (9)	0.0028 (9)	0.0069 (8)	0.0002 (9)
N1	0.0299 (9)	0.0304 (10)	0.0353 (9)	0.0037 (9)	0.0087 (7)	0.0002 (8)
N2	0.0441 (11)	0.0332 (11)	0.0362 (10)	0.0017 (9)	0.0128 (9)	0.0005 (9)

### Geometric parameters (Å, °)

**Table:**

O1—C1	1.337 (3)	C18—H18	0.9500
O1—H1	1.01 (4)	C13—C12	1.376 (4)
C5—C4	1.397 (3)	C13—C14	1.377 (4)
C5—C6	1.396 (3)	C13—H13	0.9500
C5—H5	0.9500	C10—C15	1.386 (3)
C2—C3	1.382 (3)	C10—C11	1.388 (3)
C2—C1	1.393 (3)	C10—C8	1.521 (3)
C2—H2	0.9500	C8—N1	1.477 (3)
C6—C1	1.423 (3)	C8—C9	1.520 (3)
C6—C7	1.462 (3)	C8—H8	1.0000
C4—C3	1.387 (3)	C20—C19	1.388 (4)
C4—N2	1.418 (3)	C20—H20	0.9500
C3—H3	0.9500	C19—H19	0.9500
C17—C16	1.382 (3)	C11—C12	1.389 (3)
C17—C18	1.384 (3)	C11—H11	0.9500
C17—H17	0.9500	C15—C14	1.394 (4)
C7—N1	1.279 (3)	C15—H15	0.9500
C7—H7	0.9500	C14—H14	0.9500
C16—C21	1.387 (3)	C12—H12	0.9500
C16—N3	1.445 (3)	C9—H9A	0.9800
C21—C20	1.375 (3)	C9—H9B	0.9800
C21—H21	0.9500	C9—H9C	0.9800
C18—C19	1.378 (4)	N3—N2	1.243 (3)
			
C1—O1—H1	109 (2)	C15—C10—C11	118.4 (2)
C4—C5—C6	120.1 (2)	C15—C10—C8	121.32 (19)
C4—C5—H5	119.9	C11—C10—C8	120.3 (2)
C6—C5—H5	119.9	N1—C8—C9	107.67 (17)
C3—C2—C1	119.7 (2)	N1—C8—C10	107.34 (18)
C3—C2—H2	120.1	C9—C8—C10	113.74 (18)
C1—C2—H2	120.1	N1—C8—H8	109.3
C5—C6—C1	119.3 (2)	C9—C8—H8	109.3
C5—C6—C7	120.3 (2)	C10—C8—H8	109.3
C1—C6—C7	120.3 (2)	C21—C20—C19	120.2 (2)
C3—C4—C5	119.7 (2)	C21—C20—H20	119.9
C3—C4—N2	114.5 (2)	C19—C20—H20	119.9
C5—C4—N2	125.8 (2)	C18—C19—C20	119.9 (2)
C2—C3—C4	121.3 (2)	C18—C19—H19	120.1
C2—C3—H3	119.3	C20—C19—H19	120.1
C4—C3—H3	119.3	C10—C11—C12	120.6 (2)
C16—C17—C18	119.9 (2)	C10—C11—H11	119.7
C16—C17—H17	120.1	C12—C11—H11	119.7
C18—C17—H17	120.1	C10—C15—C14	120.9 (2)
N1—C7—C6	120.3 (2)	C10—C15—H15	119.6
N1—C7—H7	119.9	C14—C15—H15	119.6
C6—C7—H7	119.9	C13—C14—C15	120.0 (2)
C17—C16—C21	120.2 (2)	C13—C14—H14	120.0
C17—C16—N3	116.2 (2)	C15—C14—H14	120.0
C21—C16—N3	123.6 (2)	C13—C12—C11	120.5 (2)
O1—C1—C2	118.4 (2)	C13—C12—H12	119.8
O1—C1—C6	121.8 (2)	C11—C12—H12	119.8
C2—C1—C6	119.8 (2)	C8—C9—H9A	109.5
C20—C21—C16	119.8 (2)	C8—C9—H9B	109.5
C20—C21—H21	120.1	H9A—C9—H9B	109.5
C16—C21—H21	120.1	C8—C9—H9C	109.5
C19—C18—C17	120.1 (3)	H9A—C9—H9C	109.5
C19—C18—H18	119.9	H9B—C9—H9C	109.5
C17—C18—H18	119.9	N2—N3—C16	112.7 (2)
C12—C13—C14	119.6 (2)	C7—N1—C8	119.7 (2)
C12—C13—H13	120.2	N3—N2—C4	115.3 (2)
C14—C13—H13	120.2		
			
C4—C5—C6—C1	−0.4 (3)	C15—C10—C8—C9	−53.4 (3)
C4—C5—C6—C7	179.25 (17)	C11—C10—C8—C9	128.4 (2)
C6—C5—C4—C3	0.8 (3)	C16—C21—C20—C19	−0.5 (4)
C6—C5—C4—N2	−177.3 (2)	C17—C18—C19—C20	0.6 (4)
C1—C2—C3—C4	1.1 (4)	C21—C20—C19—C18	0.3 (4)
C5—C4—C3—C2	−1.2 (3)	C15—C10—C11—C12	−1.7 (3)
N2—C4—C3—C2	177.2 (2)	C8—C10—C11—C12	176.6 (2)
C5—C6—C7—N1	−179.60 (19)	C11—C10—C15—C14	0.1 (3)
C1—C6—C7—N1	0.0 (3)	C8—C10—C15—C14	−178.1 (2)
C18—C17—C16—C21	1.0 (3)	C12—C13—C14—C15	−1.1 (4)
C18—C17—C16—N3	179.1 (2)	C10—C15—C14—C13	1.3 (4)
C3—C2—C1—O1	−179.6 (2)	C14—C13—C12—C11	−0.5 (4)
C3—C2—C1—C6	−0.6 (3)	C10—C11—C12—C13	1.9 (4)
C5—C6—C1—O1	179.2 (2)	C17—C16—N3—N2	172.05 (19)
C7—C6—C1—O1	−0.4 (3)	C21—C16—N3—N2	−9.9 (3)
C5—C6—C1—C2	0.3 (3)	C6—C7—N1—C8	179.21 (16)
C7—C6—C1—C2	−179.3 (2)	C9—C8—N1—C7	−107.9 (2)
C17—C16—C21—C20	−0.1 (3)	C10—C8—N1—C7	129.3 (2)
N3—C16—C21—C20	−178.1 (2)	C16—N3—N2—C4	177.16 (18)
C16—C17—C18—C19	−1.2 (4)	C3—C4—N2—N3	−174.4 (2)
C15—C10—C8—N1	65.6 (2)	C5—C4—N2—N3	3.8 (3)
C11—C10—C8—N1	−112.6 (2)		

### Hydrogen-bond geometry (Å, °)

**Table:**

*D*—H···*A*	*D*—H	H···*A*	*D*···*A*	*D*—H···*A*
O1—H1···N1i	1.01 (4)	1.66 (4)	2.557 (3)	145 (3)

Symmetry codes: i.

## References

[bb1] Akitsu, T. (2007). *Polyhedron*, **26**, 2527–2535.

[bb2] Akitsu, T. & Einaga, Y. (2005*a*). *Polyhedron*, **24**, 1869–1877.

[bb3] Akitsu, T. & Einaga, Y. (2005*b*). *Polyhedron*, **24**, 2933–2943.

[bb4] Akitsu, T. & Einaga, Y. (2006). *Acta Cryst.* E**62**, o4315–o4317.

[bb5] Akitsu, T. & Itoh, T. (2010). *Polyhedron*, **29**, 477–487.

[bb6] Akitsu, T., Takeuchi, Y. & Einaga, Y. (2004). *Acta Cryst.* C**60**, o801–o802. 10.1107/S0108270104017354 15528824

[bb7] Aslantaş, M., Kurtoğlu, N., Şahin, E. & Kurtoğlu, M. (2007). *Acta Cryst.* E**63**, o3637.

[bb8] Bruker (1998). *APEX2* and *SAINT* Bruker AXS Inc., Madison, Wisconsin, USA.

[bb9] Hadjoudis, E. & Mavridis, I. M. (2004). *Chem. Soc. Rev.* **33**, 579–588. 10.1039/b303644h 15592623

[bb10] Khandar, A. A. & Rezvani, Z. (1999). *Polyhedron*, **18**, 129–133.

[bb11] Miura, Y., Aritake, Y. & Akitsu, T. (2009). *Acta Cryst.* E**65**, o2381. 10.1107/S1600536809035557 PMC2970391 21577845

[bb12] Sheldrick, G. M. (1996). *SADABS* University of Göttingen, Germany.

[bb13] Sheldrick, G. M. (2008). *Acta Cryst.* A**64**, 112–122. 10.1107/S0108767307043930 18156677

